# Terahertz-driven linear electron acceleration

**DOI:** 10.1038/ncomms9486

**Published:** 2015-10-06

**Authors:** Emilio A. Nanni, Wenqian R. Huang, Kyung-Han Hong, Koustuban Ravi, Arya Fallahi, Gustavo Moriena, R. J. Dwayne Miller, Franz X. Kärtner

**Affiliations:** 1 Department of Electrical Engineering and Computer Science, Research Laboratory of Electronics, Massachusetts Institute of Technology, Cambridge, Massachusetts 02139, USA; 2 Center for Free-Electron Laser Science and The Hamburg Center for Ultrafast Imaging, Hamburg 22607, Germany; 3 Deutsches Elektronen Synchrotron, Ultrafast Optics and X-rays Division, Hamburg 22607, Germany; 4 Department of Chemistry and Physics, University of Toronto, Toronto, Ontario M5S, Canada; 5 Max Planck Institute for the Structure and Dynamics of Matter, Hamburg 22607, Germany; 6 Department of Physics, University of Hamburg, Hamburg 20148, Germany

## Abstract

The cost, size and availability of electron accelerators are dominated by the achievable accelerating gradient. Conventional high-brightness radio-frequency accelerating structures operate with 30–50 MeV m^−1^ gradients. Electron accelerators driven with optical or infrared sources have demonstrated accelerating gradients orders of magnitude above that achievable with conventional radio-frequency structures. However, laser-driven wakefield accelerators require intense femtosecond sources and direct laser-driven accelerators suffer from low bunch charge, sub-micron tolerances and sub-femtosecond timing requirements due to the short wavelength of operation. Here we demonstrate linear acceleration of electrons with keV energy gain using optically generated terahertz pulses. Terahertz-driven accelerating structures enable high-gradient electron/proton accelerators with simple accelerating structures, high repetition rates and significant charge per bunch. These ultra-compact terahertz accelerators with extremely short electron bunches hold great potential to have a transformative impact for free electron lasers, linear colliders, ultrafast electron diffraction, X-ray science and medical therapy with X-rays and electron beams.

## Abstract (web summary)

Pulses of light offer a way to create particle accelerators that are a fraction of the size of conventional approaches. Here, the authors demonstrate the linear acceleration of electrons with kiloelectronvolt energy gain and in extremely short bunches using optically-generated terahertz pulses.

## Graphical abstract

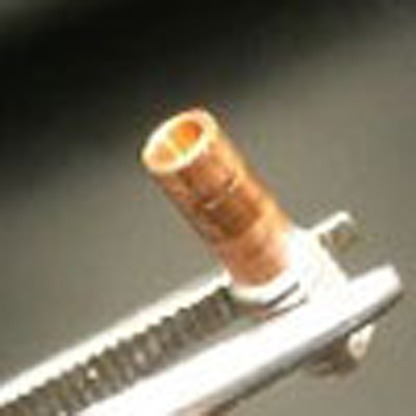

At radio-frequency (RF) frequencies where conventional sources (klystrons and so on) are efficient, surface electric field gradients in accelerating structures are limited by RF-induced plasma breakdown^1^,^2^. Empirically, the breakdown threshold has been found^3^,^4^ to scale as *E*_s_∝*f*^1/2^*τ*^−1/4^, where *E*_s_ is the surface electric field, *f* is the frequency of operation and *τ* is the pulse length indicating that higher frequencies and shorter pulse durations are clearly beneficial. This empirical scaling is limited by the onset of field emission due to the electric field on material surfaces. For most common accelerating materials, this onset is in the 10s of GV m^−1^ range^5^,^6^. In addition, low frequencies (that is, GHz) inherently require long RF pulses because a single RF cycle is long (on the order of ns) and traditional sources work most efficiently when operating over a very narrow frequency spectrum (that is, long pulse length). Practically, this results in a significant amount of average power coupled into the structure if a high repetition rate is used.

If high accelerating gradients are desired, presently available ultrafast near infrared (NIR) terawatt and petawatt laser technology based on chirped pulse amplification allows for multi-GeV m^−1^ gradients^7^,^8^,^9^,^10^,^11^,^12^,^13^,^14^,^15^,^16^. However, due to the short wavelength, infrared optical pulses prove to be difficult to use for the direct acceleration of electrons with significant charge per bunch, which is an important parameter. For example, the coherent emission in a free electron laser (FEL) from an electron bunch scales with the square of the number of electrons in the bunch^1^. To prevent emittance growth and increased energy spread, the electron bunch needs to occupy a small fraction of the optical cycle. Even for a long-infrared wavelength of 10 μm, 1° of phase in the optical wavelength corresponds to only ∼28 nm. Another practical concern would be the timing precision between the optical cycle and the arrival of the electron bunch. For example, 1° phase jitter, commonly required for operational accelerators, requires <100 as timing jitter between the optical pulse and the electron bunch, which is challenging to maintain over extended distances. Difficulties increase further when considering the available options for guiding the optical light to decrease the phase velocity to match the electron beam. A guided mode at a wavelength of 10 μm would require sub-micron precision for aligning the electron bunch and the optical waveguide.

Alternatively, laser plasma wakefield accelerators have demonstrated GeV m^−1^ accelerating gradients^7^,^9^,^17^ with 100 terawatt to petawatt (TW–PW) low repetition rate sources with percent-level energy spread and jitter for the electron bunch. The complexity of these plasma wakefield accelerators is significant because the acceleration mechanism relies on plasma bubbles, which are easily subject to instabilities. Also, power scaling of the required high-energy femtosecond pulse sources is challenging.

THz frequencies provide the best of both worlds^18^,^19^,^20^,^21^. On one hand, the wavelength is long enough that we can fabricate waveguides with conventional machining techniques, provide accurate timing and accommodate a significant amount of charge per bunch. At 0.3 THz, the wavelength is 1 mm and 1° of phase precision corresponds to 10-fs timing jitter, which is readily achievable^22^. On the other hand, the frequency is high enough that the plasma breakdown threshold for surface electric fields increases into the multi-GV m^−1^ range^6^. In addition, using optical generation techniques, we can have very short THz pulses (≤100 ps) generated by picosecond lasers readily available at high average power and under rapid development. These short pulses allow for a limited amount of pulsed heating and a limited amount of average power loading at high repetition rates (on the order of kHz and above). Both the increase in operational frequency and reduction in pulse length will play a role in increasing the breakdown limit.

Here we report the experimental demonstration of electron acceleration using the axial component of an optically generated 10 μJ THz pulse centred at 0.45 THz (see Methods: THz pulse generation) in a waveguide. The THz pulse accelerates electrons in a circular waveguide consisting of a dielectric capillary with a metal outer boundary. The dielectric slows the group and phase velocity of the THz wave allowing it to accelerate low-energy electrons. We a observe a maximum energy gain of 7 keV in 3 mm. Future, THz accelerators designed for relativistic electron beams and using more intense THz sources will be able to reach GeV m^−1^ accelerating gradients.

## Results

### Operation of the THz LINAC

**Figure 1:**
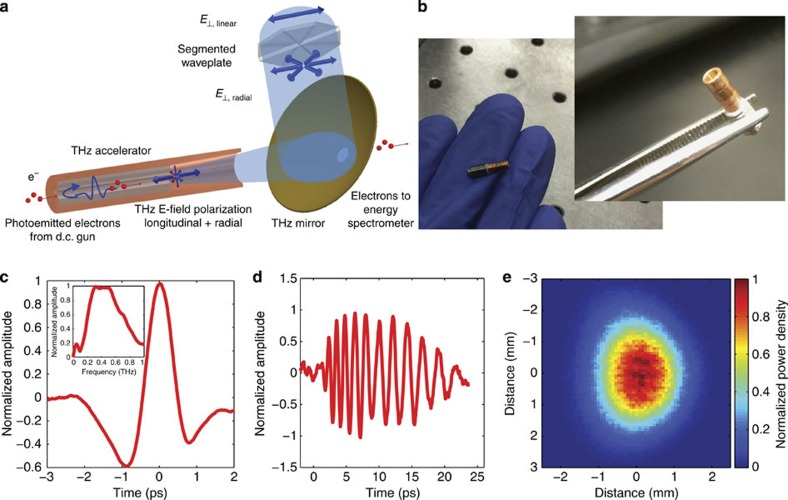
Terahertz-driven linear accelerator. (**a**) Schematic of the THz LINAC. Top right: a linearly polarized THz pulse is converted into a radially polarized pulse by a segmented waveplate before being focused into the THz waveguide. The THz pulse is reflected at the end of the waveguide to co-propagate with the electron bunch, which enters the waveguide through a pinhole (lower left). The electron bunch is accelerated by the longitudinal electric field of the co-propagating THz pulse. The electron bunch exits the THz waveguide and passes through a hole in the focusing mirror (right) for the THz pulse. (**b**) Photograph of the compact millimetre scale THz LINAC. (**c**) The time-domain waveform of the THz pulse determined with electro-optic sampling (see Methods: Electro-optic sampling). Insert: corresponding frequency-domain spectrum. (**d**) The time-domain waveform of the THz pulse at the exit of a THz waveguide 5 cm in length, including two tapers. (**e**) Normalized intensity of the focused THz beam.

A schematic view of the THz accelerator is shown in Fig. 1a with a photograph of the THz LINAC in Fig. 1b. Using 60-keV electrons, from a d.c. electron gun, an energy gain of 7 keV is observed in a 3-mm interaction length. The single-cycle THz pulse, see Fig. 1c–e, is produced via optical rectification of a 1.2 mJ, 1.03 μm laser pulse with a 1 kHz repetition rate (see Methods: Pump source). The THz pulse, whose polarization is converted from linear to radial by a segmented waveplate (see Methods: Radially polarized THz beam), is coupled into a waveguide with 10 MV m^−1^ peak on-axis electric field (see Methods: Structure testing). A 25-fC input electron bunch is produced with a 60-keV d.c. photoemitting cathode excited by a 350-fs ultraviolet pulse (see Methods: Ultraviolet photoemitter). The accelerating gradient in the THz structures demonstrated in this work can be as high as GeV m^−1^ with a single-cycle THz pulse of 10 mJ (see Methods: THz LINAC), which can be readily produced by a 250-mJ infrared pulse when using optimized THz generation^23^. Laser systems producing such and even higher energy picosecond pulses with up to kHz repetition rates are on the horizon^24^,^25^,^26^.

In this experiment, the THz waveguide supports a travelling TM_01_ mode that is phase matched to the velocity of the electron bunch produced by the d.c. photoinjector. It is the axial component of the TM_01_ mode that accelerates the electrons as they co-propagate down the waveguide. A travelling-wave mode is advantageous when considering the available single-cycle THz pulse because it does not require resonant excitation of the structure. A dielectric-loaded circular waveguide was selected due to the ease of fabrication in the THz band^27^. The inner diameter of the copper waveguide is 940 μm with a dielectric wall thickness of 270 μm. This results in a vacuum space with a radius of 200 μm. The significant thickness of the dielectric is due to the low energy of the electrons entering the structure, and will decrease significantly at higher energy. One critical aspect for THz electron acceleration is proper interaction between the electron beam and the THz pulse. Coupling the radially polarized THz pulse into the single-mode dielectric waveguide was achieved with a centrally loaded dielectric horn. The design was optimized to maximize coupling with minimal fabrication complexity. Finite element electromagnetic simulations with HFSS^28^ indicate excellent coupling of the THz pulse over a ∼200 GHz bandwidth (see Methods: Structure testing), which is compatible with the bandwidth of the radially polarized mode converter. The accelerating waveguide is 10 mm in length, including a single tapered horn for coupling the THz into the waveguide. Alignment between the THz waveguide and the d.c. gun is provided by a pin-hole aperture in a metal plate with a diameter of 100 μm that abuts the waveguide. The THz pulse is coupled into the waveguide downstream of the accelerator and it propagates along the full length of the waveguide before being reflected by the pin-hole aperture, which acts as a short at THz frequencies. After being reflected the THz pulse co-propagates with the electron bunch. The low initial energy of the electrons results in the rapid onset of a phase-velocity mismatch between the electron bunch and the THz pulse once the electrons have been accelerated by the THz pulse and this limits the interaction length to 3 mm (see Methods: THz LINAC).

### Observation of acceleration

**Figure 2:**
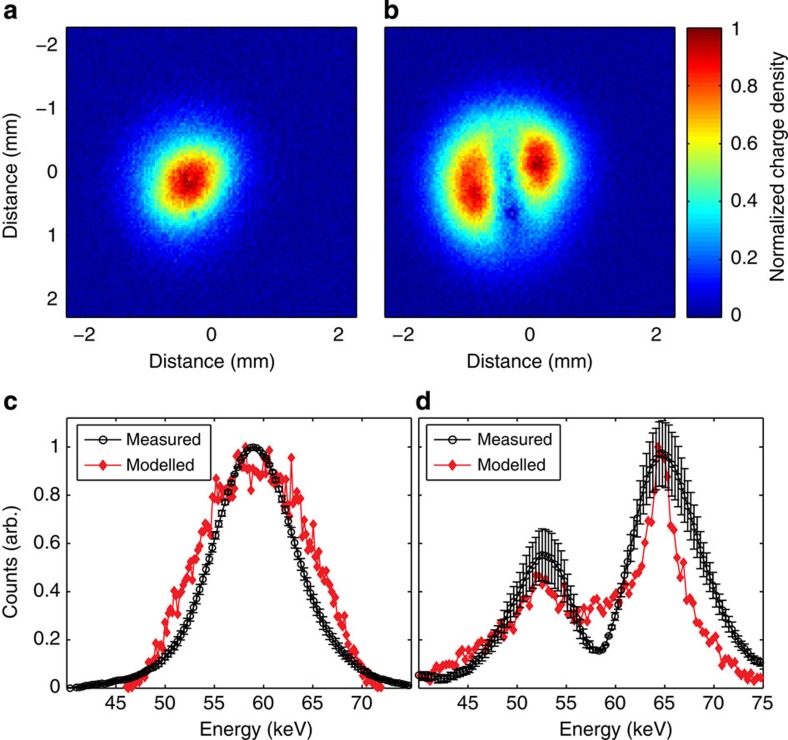
Demonstration of terahertz acceleration. Transverse electron density of the electron bunch as recorded by a micro-channel plate (MCP) at 59 kV for (**a**) THz off and (**b**) THz on. The bimodal distribution is due to the presence of accelerated and decelerated electrons, which are separated spatially by the magnetic dipole energy spectrometer. The images are recorded over a 3-s exposure at 1 kHz repetition rate. (**c**) Comparison between simulated (red) and measured (black) energy spectrum of the electron bunch measured at the MCP due to the deflection of the beam by a magnetic dipole. At 59 keV and with 25 fC per bunch, the simulation predicts a *σ*_⊥_=513 μm and Δ*E*=1.25 keV. The observed Δ*E*/*E* appears larger due to the large transverse size of the beam. After the pinhole, the transverse emittance is 25 nm rad and the longitudinal emittance is 5.5 nm rad. (**d**) Comparison between simulated (red) and measured (black) electron bunch at MCP after acceleration with THz. Decelerated electrons are present due to the long length of the electron bunch, as well as the slippage between the THz pulse and the electron bunch. Error bars in **c** and **d** correspond to one s.d. in counts over the data set of 10 integrated exposures.

The electron beam energy is determined via energy-dependent magnetic steering with a dipole located after the accelerator. Figure 2a,b shows images of the electron beam produced by the micro-channel plate detector. The measured energy spectrum from the electron bunch with and without THz is shown in Fig. 2c,d for an initial mean energy of 59 keV. The curves are compared with PARMELA^29^ particle-in-cell (PIC) simulation results used to model the d.c. gun and the THz LINAC. The full width of the electron bunch length after the pinhole is 200 μm, which is long with respect to the wavelength of the THz pulse in the waveguide, *λ*_g_=315 μm. The length of the electron bunch in combination with the phase-velocity mismatch between the electron bunch and the THz pulse results in the observation of both acceleration and deceleration of particles. With the available THz pulse energy, a peak energy gain of 7 keV was observed by optimizing the electron beam voltage and timing of the THz pulse. The modelled curve in Fig. 2d concurred with experiments for an on-axis electric field of 8.5 MV m^−1^. Using this estimated field strength, at the exit of the LINAC, the modelled transverse and longitudinal emittance are 240 and 370 nm rad, respectively. An increase in emittance from a transverse emittance of 25 nm rad and a longitudinal emittance of 5.5 nm rad after a pinhole located at the waveguide entrance is due to the long electron bunch length compared with the THz wavelength and can be easily remedied with a shorter ultraviolet pulse length.

### Optimization of electron beam interaction with THz pulse

**Figure 3:**
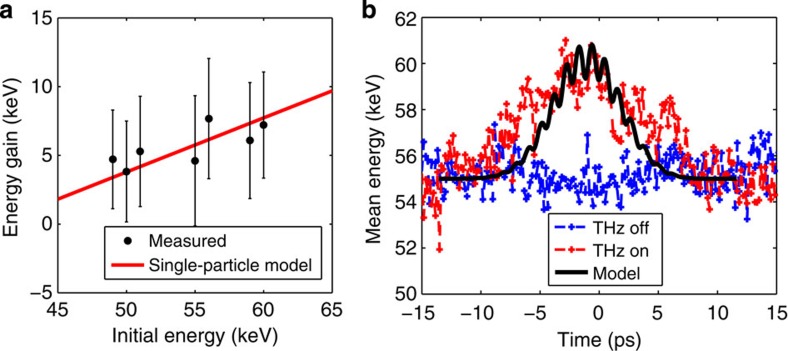
Acceleration gradient and terahertz phasing. (**a**) Scaling of energy gain for accelerated electrons as a function of the initial electron energy at the entrance of the THz LINAC. Black dots with one s.d. error bars are measured values and the red line is a single-particle model. (**b**) The temporal profile for the mean energy gain of accelerated electrons comparing the THz on and THz off signal against the simulated electron bunch. The initial electron energy was set at 55 keV to ensure stable performance of the d.c. electron gun over the acquisition time of the data set.

The energy gain achieved during the interaction of the electron bunch with the THz pulse is dependent on the initial energy of the electrons, because the set-up is operated in the non-relativistic limit where the velocity of the electrons varies rapidly. If the initial energy is decreased, the particle velocity decreases and the phase-velocity mismatch with the THz pulse increases reducing the interaction length and the acceleration of the particle. In Fig. 3a, the achieved mean output energy of the electron bunch is shown versus the initial energy. Higher initial energy was experimentally found to be favourable for higher energy gain. This observation is in agreement with a single-particle model^30^ for a peak on-axis electric field of 8.5 MV m^−1^ and an effective accelerating gradient of 2.5 MeV m^−1^ (see Methods: THz LINAC). In Fig. 3b, the mean energy of accelerated electrons is shown as a function of the THz pulse delay for 55-keV initial energy. The large temporal range of observed acceleration results from waveguide dispersion, which broadens the single-cycle pulse temporally as it propagates. Accelerated electrons are observed over the full range of the phase-matched THz pulse due to the long length of the electron bunch as it enters the THz waveguide. Modelling indicates that at the entrance of the THz waveguide for this initial energy, the electron bunch full width is 1.5 ps in length, which is already a significant fraction of the THz cycle (2.2 ps).

In conclusion, optically generated THz pulses were used to accelerate electrons in a simple and practical THz accelerator. An energy gain of 7 keV was achieved over a 3-mm interaction length with good modelled emittance. Performance of these structures improves with an increase in electron energy and gradient making them attractive for compact accelerator applications. With upgrades to pump laser energy and technological improvements to THz sources^31^, laboratory demonstration of GeV m^−1^ gradients in THz LINACs is realistic. Multi-GeV m^−1^ gradients and >10 MeV energy gain are achievable in dielectric-loaded circular waveguides^30^ with 10-mJ THz pulses and the injection of electrons at relativistic energies (see Methods: THz LINAC). The available THz pulse energy scales with infrared pump energy, with recently reported results of mJ THz pulse energies and ∼3% infrared-to-THz conversion efficiencies^32^,^33^. Multiple stages of THz acceleration can be used to achieve higher energy gain with additional infrared pump lasers for subsequent stages. Timing jitter will improve on the jitter of conventional accelerators since the accelerating field and photoemitting pulse are produced by the same drive laser. Therefore, one expects the resulting electron bunch to have tighter synchronization than possible in today's RF-based accelerators, where the photoemitting laser pulse is synchronized to the RF drive by standard RF techniques (that is, phase locked loops operating at GHz speeds). This proof-of-principle THz linear accelerator demonstrates the potential for an all-optical acceleration scheme that can be readily integrated into small-scale laboratories providing users with electron beams that will enable new experiments in ultrafast electron diffraction and X-ray production.

## Methods

### THz pulse generation

The THz pulse that is used in the accelerating structure is generated with optical rectification of 700 fs, 1.03 μm pulses in cryogenically cooled stoichiometric lithium niobate (LN) doped with 1% MgO. LN was chosen because it exhibits multiple advantages with low THz absorption, large bandgap, a high damage threshold and a high effective nonlinear coefficient, that is, large *d*_eff_. For a high efficiency of conversion in LN, a tilted-pulse-front pumping scheme^23^ is required. The THz pulse is centred at 0.45 THz with a broad spectrum ranging from 0.2 to 0.8 THz. A THz pulse energy of 10 μJ is produced from 1.2 mJ of NIR, which is slightly lower than the peak conversion efficiency of THz generation^23^ due to a larger spot size in the LN (decreased fluence) for improved transport of the THz beam. The THz beam has excellent Gaussian mode content, which allows for low-loss coupling. The THz beam was characterized spatially by a pyroelectric detector array (Spiricon Pyrocam IV, Ophir Photonics).

### Pump source

The pump source for THz generation is a Yb:KYW chirped pulse regenerative amplifier (RGA) producing 1.5-mJ pulses with 1 kHz repetition rate at a centre wavelength of 1,030 nm and bandwidth of 2.1 nm. The dielectric grating compressor following the RGA compresses the pulses to a near-transform-limited 700-fs pulse duration (full width at half maximum). The seed for the RGA was a mode-locked Yb-doped fibre oscillator emitting 70 fs, 0.2 nJ pulses at 80 MHz (ref. 34) amplified to 1.6 nJ by a Yb-doped fibre amplifier. After losses through the optical elements in the pulse front tilting set-up including a diffraction grating, the impinging pump energy into the (LN) crystal was 1.2 mJ.

### Ultraviolet photoemitter

About 2% of the available 1,030 nm NIR pump energy was used to generate the ultraviolet photoemitter pulses by fourth harmonic generation from 1.03 μm based on two-stage second harmonic generation in a 10-mm-long type I LBO crystal for infrared to green and in a 0.5-mm-long type-I BBO crystal for the green to ultraviolet, respectively. A BG-39 band-pass filter was used to remove the fundamental NIR component after the first stage. The ultraviolet pulses have a duration of ∼350 fs and are focused onto the photocathode with a beam waist of ∼200 μm. Since both the THz and ultraviolet pulses are produced from the same sub-picosecond NIR laser, the timing jitter between them is negligible.

### Electro-optic sampling

Electro-optic sampling was used to determine the temporal and spectral properties of the THz pulse and dispersion induced from the quasi-optical elements in the THz beamline. Optical synchronization between the THz pulse and the mode-locked Yb-doped fibre seed oscillator (80 MHz, 70 fs, 1,030 nm) was ensured as explained in the Pump source section of Methods. Birefringence was induced in a 200-μm thick, 110-cut ZnTe crystal. A quarter-wave plate followed by a polarizer converts the field-induced birefringence to an intensity modulation, and the intensity modulation is measured using a balanced detector with a delay scan.

### Radially polarized THz beam

The THz pulse generated by optical rectification is linearly polarized, which is not compatible with the TM_01_ mode used in the accelerating structure. A segmented half waveplate was used to convert the linearly polarized light to radially polarized light^35^. Each segment of the waveplate imparts the appropriate rotation to the polarization to transition from a linear to a radially polarized beam, which couples well in the far field to the TM_01_ mode of the accelerating structure. A segmented *λ*/2 waveplate with eight segments of ∼8-mm-thick quartz designed for operation at 0.45 THz was used.

### THz LINAC

**Figure 4:**
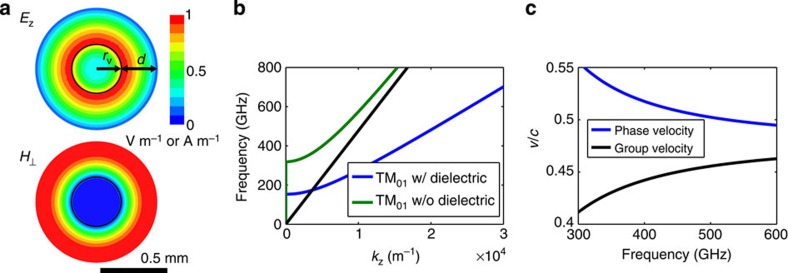
TM_01_ THz LINAC parameters. (**a**) Normalized magnitude of the longitudinal electric field and perpendicular magnetic field for the TM_01_ mode at 450 GHz in a circular copper waveguide with dielectric loading. The inner diameter of the copper waveguide is 940 μm with a vacuum radius *r*_v_=200 μm and a dielectric wall thickness of *d*=270 μm. The dimensions *r*_v_ and *d* are labelled in the *E*_z_ plot. The solid black line indicates the boundary between the vacuum core and the quartz capillary. (**b**) The dispersion relation for the TM_01_ mode with and without dielectric loading. The black line indicates the speed of light in vacuum. (**c**) The group and phase velocity of the THz pulse as a function of frequency with dielectric loading.

The THz pulse accelerates electrons in a circular waveguide consisting of a quartz capillary inserted into a hollow copper cylinder^36^, see Fig. 1a. The inner diameter of the copper waveguide is 940 μm with a dielectric wall thickness of *d*=270 μm. This results in a vacuum space with a radius of *r*_v_=200 μm. The dielectric constant of the quartz capillary is nominally *ɛ*=4.41. The operational mode of the LINAC is a travelling TM_01_ mode, see Fig. 4a. The dispersion relation for the operating mode is shown in Fig. 4b. At the centre frequency of the THz pulse (450 GHz), the group velocity is *v*_g_/*c*=0.46 and the phase velocity is *v*_p_/*c*=0.505.

Due to the operational frequency's proximity to the cutoff of the waveguide, the accelerating mode is highly dispersive with the phase and group velocity shown in Fig. 4c as a function of frequency. The waveguide dimensions of the LINAC were chosen to optimize for this experimental set-up with a low initial electron energy of 60 keV, the THz pulse energy available and the transverse dimension of the electron beam. At the nominal 60 keV, the electron velocity is *v*/*c*=0.45. This velocity is increased as the particles are accelerated increasing the electron velocity, however, the mismatch with the phase velocity results in an interaction length of 3 mm. At this point, the electron bunch and THz pulse interaction is terminated by the presence of the taper, which rapidly reduces the intensity of the on-axis electric field as the waveguide diameter is increase. This slippage causes a peak on-axis electric field of 8.5 MV m^−1^ to produce an accelerating gradient of 2.5 MeV m^−1^ at the nominal initial energy of 60 keV.

**Figure 5:**
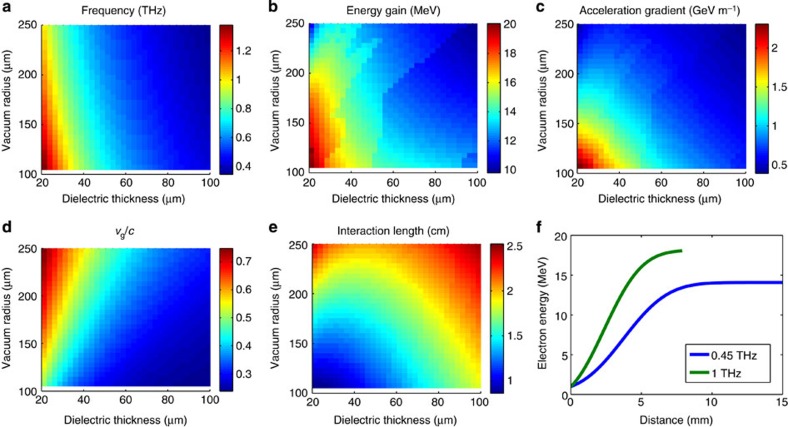
Relativistic THz LINAC design. Performance parameters as a function of vacuum radius and dielectric wall thickness for a relativistic THz LINAC operating in the TM_01_ mode with a 10 mJ single-cycle THz pulse and an initial electron energy of 1 MeV. The phase velocity is *c* for the nominal frequency of operation. The (**a**) frequency of operation, (**b**) energy gain, (**c**) acceleration gradient, (**d**) group velocity and (**e**) interaction length for the THz LINAC. (**f**) The electron energy as a function of distance for two cases, which operate with a frequency of 0.45/1 THz, a vacuum space with a radius of *r*_v_=105/105 μm and a dielectric wall thickness of 90/30 μm.

**Figure 6:**
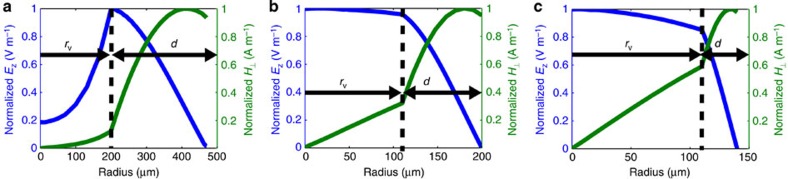
Electromagnetic field distribution. The field distribution in the THz waveguide for (**a**) the non-relativistic design described in Fig. 4 with *r*_v_=200 μm and *d*=270 μm, (**b**) the relativistic design in Fig. 5f for operation at 0.45 THz with *r*_v_=105 μm and *d*=90 μm and (**c**) the relativistic design in Fig. 5f for operation at 1 THz with *r*_v_=105 μm and *d*=30 μm.

With increased THz energy and increased electron energy, one can consider a relativistic accelerator design in which the phase velocity of the TM_01_ mode is equal to the speed of light. In addition, the design of the waveguide can be optimized to match the frequency of the available source. Figure 5a–e presents the frequency of operation, energy gain, acceleration gradient, group velocity and interaction length as a function of vacuum radius and dielectric thickness assuming a 10-mJ single-cycle THz pulse and an initial electron energy of 1 MeV. Figure 5f presents the electron energy as a function of distance for two cases, which operate with a frequency of 0.45/1 THz, a vacuum space with a radius of *r*_v_=105/105 μm and a dielectric wall thickness of *d*=90/30 μm. The drastic increase in the acceleration gradients shown in Fig. 5 is not only due to the increased THz pulse energy. The performance of a travelling-wave THz accelerator structure greatly increases when the electron velocity approaches the speed of light, because there is less dispersion for the THz pulse, a longer interaction length, the waveguide radius is decreased, the amount of dielectric material is greatly reduced and the electric field profile is improved. Figure 6 compares the field distribution for the non-relativistic waveguide design (Fig. 4), which was investigated experimentally and the relativistic designs highlighted in Fig. 5f. Note the decrease in waveguide radius, the decrease in dielectric wall thickness and the improved relative amplitude of the longitudinal electric field in the accelerating region of the waveguide (*r*<100 μm) for the two relativistic (*v*_p_=*c*) designs, which all contribute to improve the efficiency of the accelerator. The transverse fields in all cases are localized to the regions without the presence of the electron bunch alleviating concerns with the potential for ponderomotive effects. The use of multi-mJ THz pulses will increase the peak electric fields in these waveguides well above a GV m^−1^; however, the long wavelength and short propagation distance prevent the onset of nonlinearities such as self phase modulation^37^,^38^. Further details and numerical studies for relativistic THz LINACs using dielectric-loaded waveguides and few-cycle THz pulses were previously reported in ref. 30.

### Structure testing

**Figure 7:**
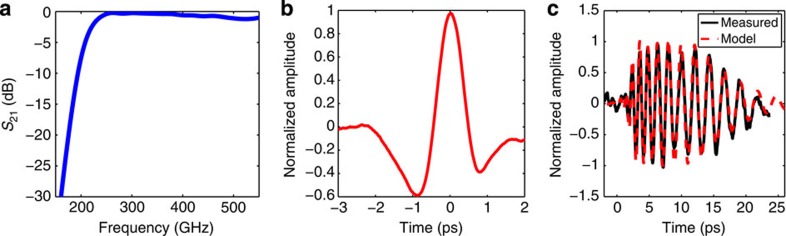
Coupling and dispersion of THz pulses in waveguides. (**a**) HFSS^28^ simulation of the coupling of the free-space radially polarized mode into the TM_01_ mode through a dielectric-loaded taper. (**b**) The time-domain waveform of the THz pulse determined with electro-optic sampling before being coupled into the THz waveguide. (**c**) The measured versus the modelled time-domain waveform of the THz pulse at the exit of a 5-cm (including tapers) dielectric-loaded THz waveguide.

A THz waveguide structure consisting of two tapers separated by a uniform waveguide section was built to test and optimize waveguide design by performing transmitted energy and polarization measurements. Measurements were performed with a Gentec-EO Pyroelectric Joulemeter Probe that is capable of measuring pulse energies exceeding 100 nJ. Efficient excitation of the TM_01_ mode, demonstrated in these measurements, is a critical requirement in developing a compact high-gradient THz LINAC. As a first step, the linearly polarized beam is converted to a radially polarized beam with a segmented waveplate. The vertical and horizontal polarization measured after the segmented waveplate were 53% and 47%, respectively. This radially polarized beam was coupled into a test waveguide structure that was 5 cm in length, including two tapers to couple the THz pulse into and out of the waveguide. The long length was selected to demonstrate that ohmic losses are manageable even over significant interaction lengths. The simulated coupling for the THz pulse into the waveguide is shown in Fig. 7a with a bandwidth of over 200 GHz. The power coupled through the structure was measured with and without dielectric loading at 32% and 54%, respectively. The increased losses in the dielectric-loaded waveguide are due to increased ohmic losses. For the dielectric-loaded waveguide, 2 μJ of energy was measured at the exit of the waveguide, which corresponds to a calculated peak on-axis electric field is 9.7 MV m^−1^.

Electro-optic sampling (see Methods: Electro-optic sampling) was used to characterize the linear dispersion of the dielectric-loaded waveguide test structure, which has the same dimensions as the waveguide used in the acceleration experiment and the properties shown in Fig. 4b,c. Figure 7b shows the time-domain spectrum produced by the THz source (see Methods: THz pulse generation). Figure 7c shows a comparison between the measured time-domain spectrum of THz pulse at the exit of the waveguide with the simulated propagation of the input THz pulse propagated with the dispersive parameters of the waveguide design provided in Fig. 4b,c. Excellent agreement indicates that the fabricated structure has the desired performance.

## Additional information

**How to cite this article:** Nanni, E. A. *et al.* Terahertz-driven linear electron acceleration. *Nat. Commun.* 6:8486 doi: 10.1038/ncomms9486 (2015).
